# Identifying *KCNJ*5 Mutation in Aldosterone-Producing Adenoma Patients With Baseline Characteristics Using Machine Learning Technology

**DOI:** 10.1016/j.jacasi.2023.03.010

**Published:** 2023-06-13

**Authors:** Li-Chin Chen, Wei-Chieh Huang, Kang-Yung Peng, Ying-Ying Chen, Szu-Chang Li, Siti Khadijah Syed Mohammed Nazri, Yen-Hung Lin, Liang-Yu Lin, Tse-Min Lu, Jung Hee Kim, Elena Aisha Azizan, Jinbo Hu, Qifu Li, Jeff S. Chueh, Vin-Cent Wu, TAIPAI Study Group

**Affiliations:** a Research Center for Information Technology Innovation, Academia Sinica, Taipei, Taiwan; b Division of Cardiology, Department of Internal Medicine, Taipei Veterans General Hospital, Taipei, Taiwan; c School of Medicine, National Yang Ming Chiao Tung University, Taipei, Taiwan; d Department of Internal Medicine, National Taiwan University Hospital, Taipei, Taiwan; e Division of Nephrology, Department of Internal Medicine, MacKay Memorial Hospital, Taipei, Taiwan; f Graduate Institute of Clinical Medicine, College of Medicine, National Taiwan University Hospital, Taipei, Taiwan; g Department of International Business, National Taipei University of Business, Taipei, Taiwan; h Department of Medicine, The National University of Malaysia (UKM) Medical Centre, Selangor, Malaysia; i TAIPAI, Taiwan Primary Aldosteronism Investigation Study Group, Taiwan; j Division of Endocrinology and Metabolism, Department of Internal Medicine, Taipei Veterans General Hospital, Taipei, Taiwan; k Healthcare and Management Center, Taipei Veterans General Hospital, Taipei, Taiwan; l Division of Endocrinology and Metabolism, Department of Internal Medicine, Seoul National University Hospital, Seoul, Korea; m Endocrine Unit, Faculty of Medicine, The National University of Malaysia (UKM) Medical Centre, Cheras, Kuala Lumpur, Malaysia; n Division of Endocrinology, the First Affiliated Hospital, Chongqing Medical University, Chongqing, China; o Glickman Urological and Kidney Institute, and Cleveland Clinic Lerner College of Medicine, Cleveland Clinic, Cleveland, Ohio; p Department of Urology, National Taiwan University Hospital, Taipei, Taiwan; q Primary Aldosteronism Center at National Taiwan University Hospital, Taipei, Taiwan

**Keywords:** *KCNJ*5 mutation, machine learning, prediction model, primary aldosteronism

## Abstract

**Background:**

Primary aldosteronism is characterized by inappropriate aldosterone production, and unilateral aldosterone-producing adenoma (uPA) is a common type of PA. *KCNJ*5 mutation is a protective factor in uPA; however, there is no preoperative approach to detect *KCNJ*5 mutation in patients with uPA.

**Objectives:**

This study aimed to provide a personalized surgical recommendation that enables more confidence in advising patients to pursue surgical treatment.

**Methods:**

We enrolled 328 patients with uPA harboring *KCNJ*5 mutations (n = 158) or not (n = 170) who had undergone adrenalectomy. Eighty-seven features were collected, including demographics, various blood and urine test results, and clinical comorbidities. We designed 2 versions of the prediction model: one for institutes with complete blood tests (full version), and the other for institutes that may not be equipped with comprehensive testing facilities (condensed version).

**Results:**

The results show that in the full version, the Light Gradient Boosting Machine outperformed other classifiers, achieving area under the curve and accuracy values of 0.905 and 0.864, respectively. The Light Gradient Boosting Machine also showed excellent performance in the condensed version, achieving area under the curve and accuracy values of 0.867 and 0.803, respectively.

**Conclusions:**

We simplified the preoperative diagnosis of *KCNJ*5 mutations successfully using machine learning. The proposed lightweight tool that requires only baseline characteristics and blood/urine test results can be widely applied and can aid personalized prediction during preoperative counseling for patients with uPA.

Primary aldosteronism (PA, or Conn’s syndrome) is characterized by the inappropriate production of aldosterone, and its prevalence is as high as 11.2% in newly diagnosed hypertensive patients, depending on the screening stringency and population.^1^^,^^2^ Unilateral aldosterone-producing adenoma (uPA) is a common type of PA^3^ and a curable form of hypertension.

Recent studies have shown that somatic mutations play a crucial role in the pathogenesis of uPA.^4^ The *KCNJ*5 somatic mutation is associated with an almost 6-fold increase in the chance of complete clinical success after adrenalectomy in patients with uPA. Furthermore, the presence of the *KCNJ*5 somatic mutation was an independent hypertension remission predictor after unilateral adrenalectomy in patients with uPA.^5^ Presently, the identification of *KCNJ*5 mutations requires adrenalectomy and sampling of adrenal tumor tissues for Sanger sequencing. This procedure is invasive, highly complex, and enables accurate diagnosis after adrenalectomy. However, the comprehensive data required to ensure surgical indication also necessitates access to highly sophisticated medical equipment, making this method impractical for general practitioners. Regarding the development of a prediction method using clinical values commonly used in PA diagnostic steps, studies using machine learning for uPA are beginning to be seen recently.^6^, ^7^, ^8^ However, there are currently no associated results on the detection of *KCNJ5-mutated* uPA.^9^ Therefore, it is important to find a general approach to predict *KCNJ5* mutations in patients with uPA. We aimed to develop a machine learning model to forecast the occurrence of *KCNJ*5 mutations in patients with uPA, using baseline demographic characteristics and laboratory data. This emphasizes the clinical utility of personalized therapy during preoperative counseling for blood pressure responses.

## Methods

### Study design and participants

**Figure 1:**
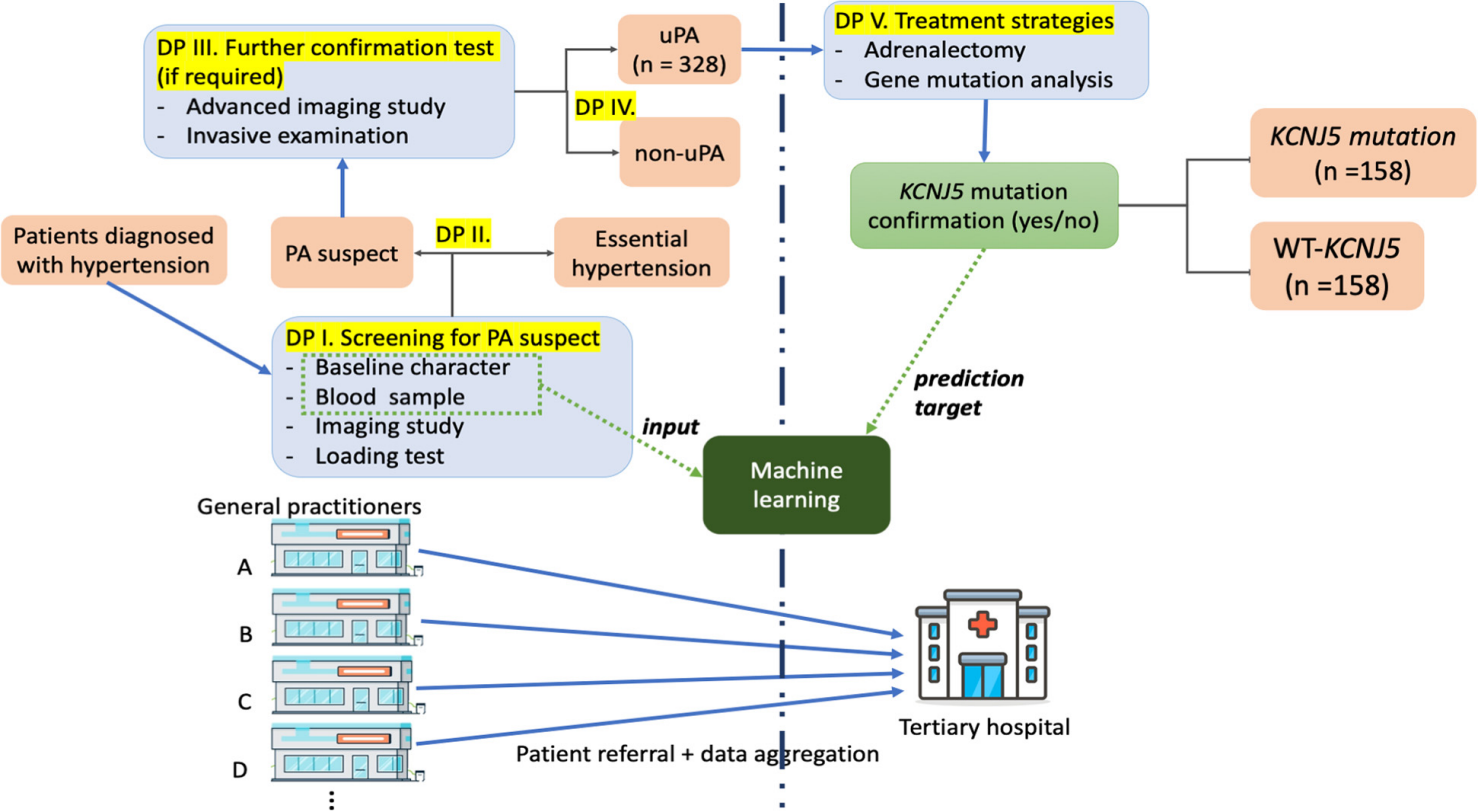
The Patient Recruitment Flow In the general hospital, patients diagnosed with hypertension and suspected primary aldosteronism (PA) underwent screening for suspected PA, illustrated as decision point (DP) I. Patients with an initial aldosterone-renin ratio (ARR) of >35 were confirmed to have PA through saline infusion or captopril tests (DP II). Subtypes of PAs (unilateral primary aldosteronism [uPA] and non-uPA) were identified by advanced imaging studies or other invasive examinations if required (DP III). Identifying uPA requires the criteria to be satisfied (DP IV) and the criteria was listed in Supplemental Table 1. Standard TAIPAI protocol and Aldosteronism Consensus in Taiwan. After confirming the PA subtypes, patients were transferred to a tertiary hospital for adrenalectomy and gene mutation analysis.

The inception cohort was based on the Taiwan Primary Aldosteronism Investigation (TAIPAI) database and the tissue bank and associated information is provided in the Supplemental Appendix.^10^ A patient recruitment flowchart is shown in Figure 1 and all enrolled patients need to fulfill all criteria. In the general hospital, patients diagnosed with hypertension and suspected PA underwent screening for suspected PA, illustrated as decision point (DP) I in Figure 1. Before PA confirmation screening, antihypertensive medications were discontinued for at least 21 days before conducting confirmatory tests.^11^ Patients with an initial aldosterone-renin ratio of >35 were confirmed to have PA through saline infusion or captopril tests (DP II). Subtypes of PAs (uPA and non-uPA) were identified by further examination, including advanced imaging studies or other invasive examinations if required (DP III). Identifying uPA requires the following criteria to be satisfied^11^ (DP IV): 1) PA diagnosis was confirmed; 2) imaging evidence was available for a unilateral adrenal adenoma or hyperplasia; 3) lateralization of aldosterone hypersecretion with adrenal vein sampling or during dexamethasone suppression NP-59 single-photon emission computed tomography^12^ to the abovementioned imaging findings; or 4) uPA was further confirmed after adrenalectomy with a pathologically proven CYP11B2-positive stained adenoma or through immunohistochemical evidence for (multiple) aldosterone-producing micronodule(s) after adrenalectomy.^13^ After confirming the PA subtypes, patients were transferred to a tertiary hospital for adrenalectomy and gene mutation analysis. If the previous institute was unable to determine the subtypes of PA, the patients were transferred to a tertiary hospital for advanced imaging or invasive examination. The study complied with the Declaration of Helsinki and was approved by the institutional review board of the National Taiwan University Hospital, Taipei, Taiwan (No. 200611031R). All participants signed an informed consent form before their inclusion in the study. All experiments were performed in accordance with the approved guidelines.

### Adenoma after adrenalectomy

In each case, unilateral adrenalectomy of the lesion-side adrenal gland was performed by experienced surgeons using a lateral transperitoneal laparoscopic approach. Excised adrenal tumors were frozen immediately and stored at −80 °C. Conventional somatic mutation hotspots of *KCNJ5* in DNA samples from representative adenoma tissue sections with the largest circumscribed encapsulated tumor areas and a distinctive golden-yellow cut surface were sequenced using Sanger sequencing.

### Laboratory measurements

Plasma aldosterone concentration and plasma renin activity were evaluated using commercial radioimmunoassay kits: the ALDO-RIACT RIA kit (Cisbio Bioassays) and the GammaCoat DiaSorin, respectively.^14^ The potassium level below 3.5 mmol/L was defined as hypokalemia.^15^

### KCNJ5 sequencing and mutation analysis

For mutation analysis, the entire coding region of the KCNJ5 gene was amplified by polymerase chain reaction with gene-specific primers and then by Sanger sequencing with the BigDye Terminator v.3.1 Cycle Sequencing Kit (Applied Biosystems Inc), as previously reported.^16^ Sequence analysis was performed using DNAStar Lasergene SeqMan Pro 7.1.0 software (DNAStar Inc).

### Development of predictive models

**Figure 2:**
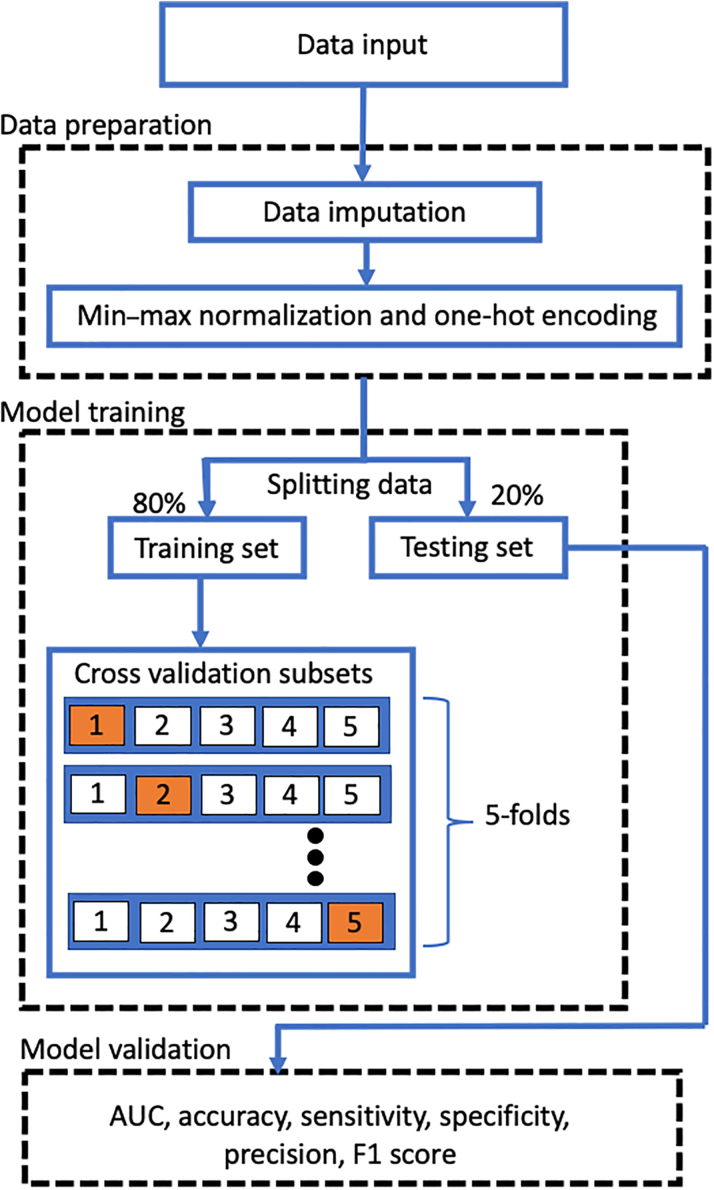
Data Processing and Model Training Flow The model training process consists of 3 stages: data preparation, model training, and model validation. During the data preparation stage, data imputation and feature normalization were performed. Regarding model training, we tested multiple machine learning algorithms to find the classifier with the best performance. Finally, in the model validation stage, the model was evaluated based on the testing dataset, which was isolated at the beginning of the training process and considered the unseen data. The evaluation metrics included the receiver operating characteristic (ROC) curve, the area under the curve (AUC), accuracy, sensitivity, specificity, precision, and the F1 score.

Two application scenarios are designed. For institutes with comprehensive laboratory capacities, we trained the model based on the full version of the examinations, in which 87 baseline and laboratory parameters were used to predict *KCNJ*5 mutations. For institutes that may not have been equipped with comprehensive equipment, a condensed version was designed to simplify the required parameters. For this, we used statistical tests and feature importance ranking as approaches to reduce the parameter requirements. The robustness of the model was based on its general performance in all aspects. Therefore, the evaluation metrics (including area under the curve [AUC], accuracy, sensitivity, specificity, and F1) were averaged, and an average score was calculated to compare the approaches. The model training process is illustrated in Figure 2, and the detailed development information is described in the Supplemental Appendix.

Further, due to the characteristics of uPA, we trained additional versions to investigate the impacts of character-specific variables, such as sex and hypokalemia, among patients with uPA. In addition, to demonstrate the robustness of the model, we trained an extremely condensed version that complied with a Malaysian dataset and performed external validation. The details of the model designs of the condensed version, character-specific version, extremely condensed version, and external validation are illustrated in the Supplemental Appendix.

### Statistical analysis

All continuous data are presented as mean ± SD or 95% CIs. Differences in continuous data between the 2 groups were compared using a 2-sample *t*-test. Categorical data between the 2 groups were compared using chi-square or Fisher exact tests. Statistical significance was set at *P* < 0.05. All statistical analyses were performed using SPSS statistical software (IBM SPSS Statistics for Windows, version 22.0).

## Results

In total, 328 patients were enrolled; there was a mild predominance of women (53%) among the participants, with an average age of 51.3 ± 11.6 years (Table 1). The 87 parameters collected in this study, including routine blood and urine tests, clinical demographics, and comorbidities, are presented in Table 1 and Supplemental Table 1. The parameters that showed statistically significant differences between the 2 groups of patients with uPA are listed in Table 1 and those with no statistically significant differences are listed in Supplemental Table 1. Based on the statistical results (Supplemental Tables 2 and 3), we found that patients with uPA harboring *KCNJ*5 mutations were significantly younger, had smaller waistlines, a higher prevalence of hypokalemia, and shorter hypertension durations. Serum sample analysis showed that patients harboring *KCNJ*5 mutations had higher aldosterone, sodium, bicarbonate, 8 am adrenocorticotropic hormone, and intact parathyroid hormone levels and lower blood urea nitrogen, creatinine, potassium, lowest serum potassium, calcium, urine acid, glucose, insulin, cholesterol, and triglyceride levels. However, in urine sample analysis, those harboring *KCNJ*5 mutations had higher levels of 24-h urine aldosterone and transtubular potassium gradients and lower levels of creatinine, sodium, chloride, and osmolality compared with those without the mutation.

**Table 1:** Statistically Significant Differences Among Parameters of Operated Unilateral Primary Aldosteronism Patients Harboring *KCNJ*5 Mutation or Not

	All (N = 328)	*WT-KCNJ5* (n = 158)	*KCNJ5* mutations (n = 170)	*P* Value
Baseline characteristics				
Age (y)	51.30 ± 11.6	54.70 ± 11.6	48.35 ± 10.6	<0.001
Waistline (cm)	83.43 ± 11.5	86.20 ± 12.0	81.02 ± 10.7	<0.001
Comorbidities				
Hypokalemia (n)	52 (15.9)	11 (7.0)	41 (24.1)	<0.001
Hypertension (n)	315 (96.0)	148 (93.6)	167 (98.2)	0.023
Latency of HTN (y)	7.52 ± 7.14	8.68 ± 8.26	6.54 ± 6.44	<0.001
Laboratory data				
Serum aldosterone (ng/dL)	58.41 ± 48.5	51.66 ± 46.1	65.09 ± 49.9	0.012
Serum BUN (ng/dL)	14.34 ± 4.9	15.13 ± 5.1	13.61 ± 4.7	0.010
Serum creatinine (mg/dL)	0.91 ± 0.3	0.94 ± 0.4	0.87 ± 0.3	0.035
Serum Na^+^(mg/dL)	140.45 ± 8.3	139.96 ± 3.2	141.73 ± 2.9	<0.001
Serum K^+^ (mmol/L)	3.47 ± 0.66	3.77 ± 0.52	3.20 ± 0.65	<0.001
Lowest serum K^+^ (mmol/L)	3.27 ± 0.68	3.60 ± 0.62	2.96 ± 0.58	<0.001
Serum Ca^2+^ (mmol/L)	2.29 ± 0.41	2.30 ± 0.11	2.24 ± 0.20	0.002
Serum UA (mmol/L)	5.79 ± 1.52	6.05 ± 1.58	5.55 ± 1.44	0.007
Serum glucose (mg/dL)	101.83 ± 24.57	104.90 ± 26.42	98.94 ± 22.38	0.034
Serum insulin (μIU/ml)	11.13 ± 11.3	12.99 ± 12.7	9.43 ± 9.61	0.029
Serum cholesterol (mg/dL)	184.02 ± 38.6	188.77 ± 42.6	179.72 ± 34.2	0.050
Serum triglyceride (mg/dL)	120.13 ± 71.9	139.87 ± 86.14	101.87 ± 49.5	<0.001
Serum pH	7.41 ± 0.04)	7.40 ± 0.05	7.42 ± 0.04	0.004
Serum HCO_3_^−^ (mmol/L)	26.81 ± 5.17	24.96 ± 4.17	28.38 ± 5.46	<0.001
8 am serum ACTH (pg/mL)	18.87 ± 14.15	16.89 ± 13.20	21.20 ± 14.92	0.023
Serum i-PTH (pg/mL)	74.23 ± 41.02	66.01 ± 36.45	81.92 ± 47.58	0.024
Urine aldosterone (μg/24 hrs)	19.16 ± 15.71	16.06 ± 14.39	21.77 ± 16.35	0.010
Urine creatinine (mg/dL)	75.28 ± 47.27	82.38 ± 52.68	68.89 ± 41.00	0.025
Urine Na^+^ (mmol/L)	68.50 ± 34.63	75.98 ± 38.34	61.06 ± 28.03	0.001
Urine Cl^−^ (mmol/L)	70.48 ± 29.35	75.04 ± 30.62	65.24 ± 25.63	0.023
Urine osmolarity (mOsm/Kg)	380.76 ± 143.86	402.48 ± 163.13	363.18 ± 124.41	0.042
TTKG	6.34 ± 2.88	5.41 ± 2.15	7.14 ± 3.19	<0.001

Values are mean ± SD or n (%).

ACTH = adrenocorticotropic hormone; BUN = blood urea nitrogen; HTN = hypertension; i-PTH = intact parathyroid hormone; TTKG = transtubular potassium gradient; UA = uric acid; WT = wild-type.

**Figure 3:**
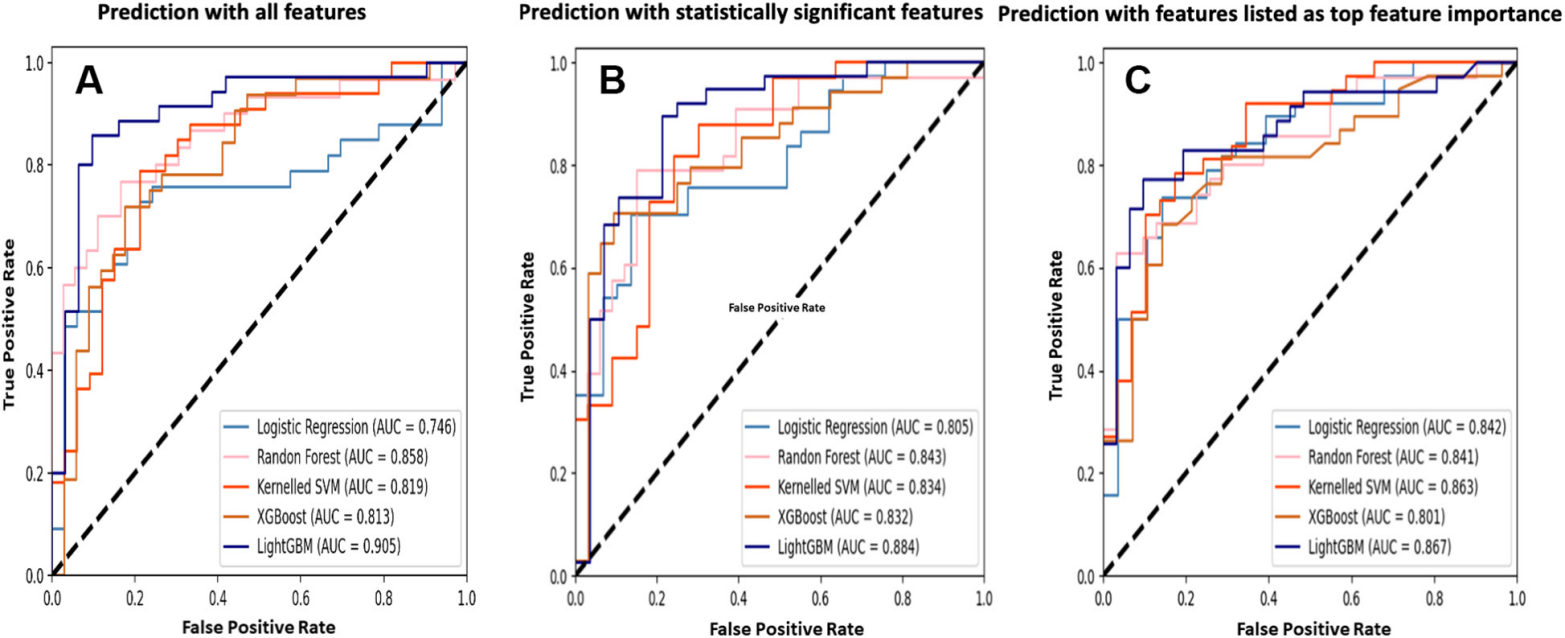
ROC Curves of Classifiers in Each Version Receiver operating characteristic (ROC) curves for each machine learning classifier in the full and condensed versions are shown. The results showed that in the full examination version **(A)**, Light Gradient Boosting Machine (LightGBM) outperformed the other classifiers, with the highest performance for all indicators (AUC = 0.905; accuracy = 0.864; average score = 0.871). Meanwhile, 27 parameters showed significant differences between patients harboring *KCNJ5* mutations and wild-type (WT)-*KCNJ*5 carriers. Therefore, we also selected 27 features that were listed as having the highest ranking in terms of feature importance by LightGBM. **(B)** and **(C)** show the corresponding ROC curves for each feature selection method.

**Figure 4:**
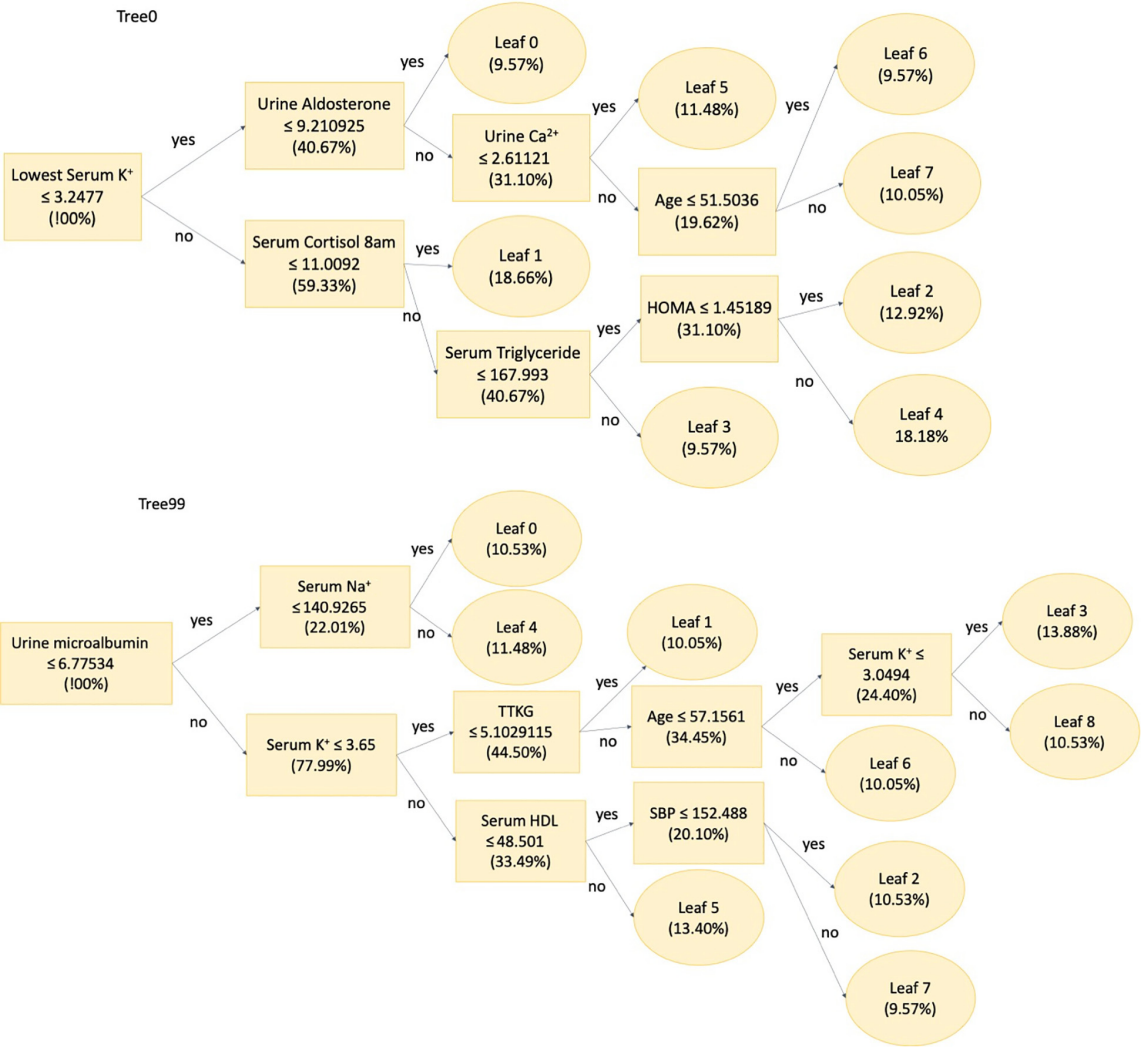
Two Sampled Trees From LightGBM The figure shows the 2 decision trees (among a total of 100 trees) that were used in the prediction model and decision rules. In general, 100 trees comprised 785 classification rules. HDL = high-density lipoprotein; HOMA = homeostatic model assessment; SBP = systolic blood pressure; TTKG = transtubular potassium gradient.

Table 2 shows the predictive performances for each machine learning classifier in the full and condensed versions, along with the receiver operating characteristic curves corresponding to each classifier (Figure 3). The results showed that in the full examination version (Table 2, Figure 3A), Light Gradient Boosting Machine (LightGBM) outperformed the other classifiers, with the highest performance for all indicators (AUC = 0.905; accuracy = 0.864; average score = 0.871). Figure 4 shows the 2 decision trees (among a total of 100 trees) that were used in the prediction model and decision rules. In general, 100 trees comprised 785 classification rules.

**Table 2:** Predictive Performance for Each Classifier

	LightGBM	XGBoost	Kernelled SVM	Random Forest	Logistic Regression
Predictive performance for each classifier					
AUC	0.905 (0.858–0.980)	0.813 (0.733–0.904)	0.819 (0.776–0.920)	0.858 (0.716–0.887)	0.746 (0.631–0.861)
Accuracy	0.864 (0.788–0.924)	0.742 (0.659–0.849)	0.758 (0.697–0.856)	0.758 (0.613–0.803)	0.742 (0.598–0.818)
Sensitivity	0.886 (0.779–0.973)	0.719 (0.586–0.879)	0.727 (0.613–0.834)	0.767 (0.603–0.850)	0.758 (0.554–0.875)
Specificity	0.839 (0.716–0.949)	0.765 (0.662–0.884)	0.788 (0.727–0.940)	0.750 (0.557–0.873)	0.727 (0.515–0.844)
Precision	0.861 (0.743–0.954)	0.742 (0.671–0.893)	0.774 (0.729–0.937)	0.719 (0.588–0.875)	0.735 (0.582–0.870)
F1	0.873 (0.780–0.932)	0.730 (0.639–0.864)	0.750 (0.676–0.851)	0.742 (0.621–0.824)	0.746 (0.621–0.841)
Average	0.871	0.752	0.769	0.766	0.742
Condensed version using statistically significant features (number of features = 27)					
AUC	0.884 (0.869–0.959)	0.832 (0.734–0.930)	0.834 (0.758–0.931)	0.843 (0.736–0.915)	0.805 (0.717–0.914)
Accuracy	0.773 (0.750–0.887)	0.773 (0.652–0.856)	0.773 (0.697–0.864)	0.742 (0.667–0.818)	0.712 (0.652–0.833)
Sensitivity	0.737 (0.675–0.919)	0.706 (0.606–0.873)	0.788 (0.639–0.914)	0.788 (0.629–0.891)	0.757 (0.626–0.906)
Specificity	0.821 (0.714–0.939)	0.844 (0.607–0.879)	0.758 (0.637–0.932)	0.697 (0.629–0.880)	0.655 (0.582–0.845)
Precision	0.848 (0.716–0.934)	0.828 (0.641–0.887)	0.765 (0.693–0.934)	0.722 (0.648–0.896)	0.737 (0.590–0.881)
F1	0.789 (0.733–0.901)	0.762 (0.630–0.859)	0.776 (0.680–0.886)	0.754 (0.662–0.838)	0.747 (0.617–0.844)
Average	0.809	0.791	0.782	0.758	0.736
Condensed version using top features importance measurement ranking (number of features = 27)					
AUC	0.867 (0.839–0.965)	0.801 (0.701–0.903)	0.863 (0.729–0.922)	0.841 (0.724–0.898)	0.842 (0.743–0.903)
Accuracy	0.803 (0.758–0.909)	0.758 (0.636–0.833)	0.773 (0.652–0.826)	0.742 (0.652–0.833)	0.758 (0.689–0.848)
Sensitivity	0.829 (0.742–0.944)	0.684 (0.542–0.800)	0.811 (0.583–0.894)	0.714 (0.605–0.882)	0.737 (0.656–0.912)
Specificity	0.774 (0.691–0.922)	0.857 (0.654–0.906)	0.724 (0.611–0.886)	0.774 (0.600–0.874)	0.786 (0.600–0.851)
Precision	0.806 (0.737–0.939)	0.867 (0.608–0.897)	0.789 (0.619–0.902)	0.781 (0.642–0.877)	0.824 (0.635–0.881)
F1	0.817 (0.748–0.919)	0.765 (0.606–0.820)	0.800 (0.656–0.837)	0.746 (0.651–0.857)	0.778 (0.661–0.867)
Average	0.816	0.789	0.793	0.766	0.788

Values are validation results (95% CI).

AUC = area under the curve; LightGBM = Light Gradient Boosting Machine; SVM = support vector machine; XGBoost = extreme Gradient Boosting.

**Figure 5:**
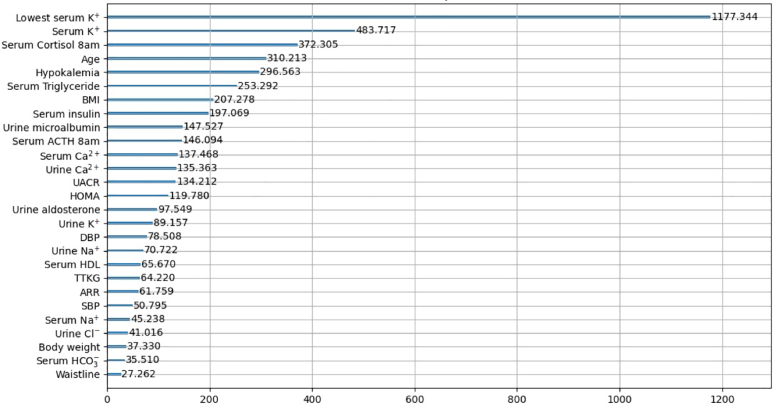
Top 27 Feature Importance Rankings The figure shows a visualized plot of the feature importance ranking. The general performance of feature importance (average score = 0.816) outperformed the statistically significant features (average score = 0.809). This indicates that in the condensed version, feature importance measurement may be a more effective approach for LightGBM (AUC = 0.867; accuracy = 0.803) to perform better predictions compared with the statistical approach; it generally outperformed the other methods. The top 27 ranked important features of the other algorithms are also listed in Supplemental Table 5. ACTH = adrenocorticotropic hormone; ARR = aldosterone-renin ratio; BMI = body mass index; DBP = diastolic blood pressure; UACR = urine albumin creatinine ratio; other abbreviations as in Figure 4.

Meanwhile, 27 parameters showed significant differences between patients harboring *KCNJ5* mutations and wild-type *KCNJ*5 carriers. Therefore, we also selected 27 features that were listed as having the highest ranking in terms of feature importance by LightGBM. The prediction results of the condensed version are shown in Table 2 and Figures 3B and 3C show the corresponding receiver operating characteristic curves for each feature selection method. Supplemental Table 4 lists the full features and 2 selected features, and Figure 5 shows a visualized plot of the feature importance ranking. The general performance of feature importance (average score = 0.816) outperformed the statistically significant features (average score = 0.809). This indicates that in the condensed version, feature importance measurement may be a more effective approach for LightGBM (AUC = 0.867; accuracy = 0.803) to perform better predictions compared with the statistical approach; it generally outperformed the other methods. The top 27 ranked important features of the other algorithms are listed in Supplemental Table 5.

The disease character-specific investigations are shown in the Supplemental Appendix and Supplemental Table 6. In our study, 276 (84.1%) patients had normokalemia, and it appeared that the model performance degraded when trained with patients with pure normokalemia. Hypokalemia is an important parameter that is listed as fifth in the full examination version. Meanwhile, the results of the sex-specific analysis showed that the performance generally did not differ significantly; however, the female-specific model performed less desirably in specificity, implying that the model tended to misclassify patients without the mutation. The performance of the extremely condensed version managed to approximate the condensed version, and the result of Malaysian external validation slightly decreased (average score = 0.733) (Supplemental Table 6 to 8).

## Discussion

The results show that in the full version, the LightGBM outperformed the other classifiers, achieving AUC and accuracy values of 0.905 and 0.864, respectively. LightGBM also showed excellent performance in the condensed version, achieving AUC and accuracy values of 0.867 and 0.803, respectively. This condensed model was also validated by an external Malaysia dataset and showed good performance. Our work serves as an alternative for *KCNJ*5 mutation identification; therefore, the predictive model potentially broadened the use of personalized assessment and increased the number of patients who underwent the recommended surgical treatment. Moreover, patients without *KCNJ*5 mutations should be followed up carefully after adrenalectomy to prevent a high possibility of treatment failure (Central Illustration).

**Central Illustration:**
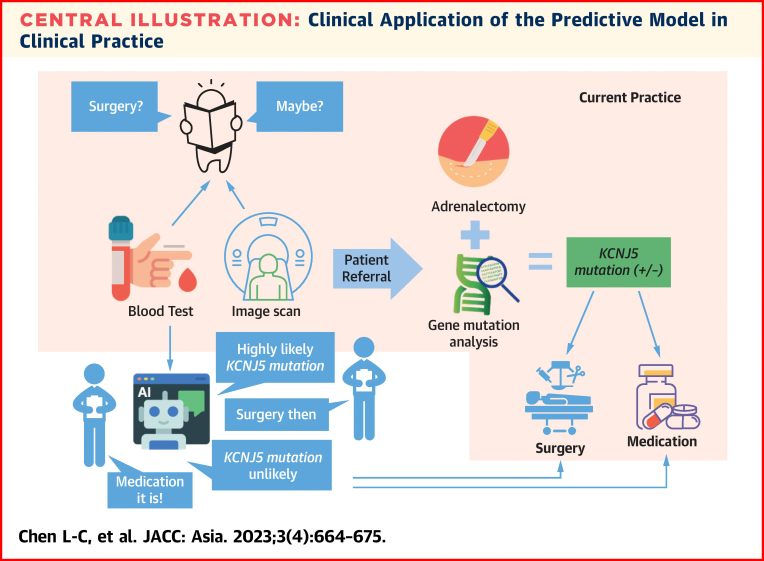
Clinical Application of the Predictive Model in Clinical Practice In clinical practice, the patents received blood test, confirmation test, subtypes differentiation, and advancing image scan. Then, we will suggest treatment strategies based on previous finding of examination. After adrenalectomy, we will perform gene mutation analysis; however, most patients hesitated to receive adrenalectomy. Our predictive model can potentially aid in providing better surgical options for patients with unilateral aldosterone-producing adenoma (uPA) who could benefit from ipsilateral adrenalectomies and guide postoperative strategy.

### The features of full and condensed versions

In this study, we found that our full and condensed versions were more capable of identifying *KCNJ*5 mutation carriers in patients with uPA. The performance of the model decreased when the features were reduced. Nevertheless, the condensed version maintained an accuracy above 80%. Regarding potassium-related features and serum sodium levels, serum potassium and sodium levels were highlighted in our model, which could reflect the physiological importance of aldosterone in regulating renal potassium and sodium levels.^17^ In clinical practice, the potassium level is regarded as a key feature in patients with uPA. However, serum lipid metabolism and serum calcium levels were also helpful in identifying *KCNJ*5 mutation carriers, consistent with previous reports suggesting that patients with uPA have significantly lower levels of serum triglycerides, serum cholesterol, and secondary hyperparathyroidism than those with bilateral PA.^18^^,^^19^ The relationship between serum aldosterone overproduction and serum lipid metabolic disturbances in patients with uPA was not revealed in this study. In the TAIPAI group, Chen et al^20^ evaluated the prevalence of metabolic disorders and abdominal obesity, showing that patients with uPA who harbored *KCNJ5* mutations had a significantly lower prevalence of metabolic syndrome and a lower distribution of body adipose tissue. They also found that patients who were *KCNJ*5 mutation carriers had significantly lower serum triglyceride and cholesterol levels, suggesting that different mechanisms, including adipocyte renin-angiotensin system dysregulation, may be involved. The main findings of these studies were consistent with those of this study. However, the detailed mechanism remains unclear and further research is required to address these issues. Although the extremely condensed version managed to approximate the average score as the condensed version, the chosen parameters were limited owing to local practice limitations, which were not based on systematic selection and validation. Therefore, we recommend a minimum of 27 blood test parameters for accurate *KCNJ5* mutation diagnosis.

### Technology-aided practices and methodology details

We opted to develop a tool that uses machine learning technology rather than traditional scoring-based measurements. Unlike traditional scoring measurements, the classification thresholds determined by machine learning models naturally handle complexity. In this study, each case underwent classification using 100 trees (accumulating 785 rules in total), and we obtained a probability score for each patient that led to the final decision. This enabled a more sophisticated and specific assessment for diagnosing patients while reducing the burden of patient examination and all-items collection compared with traditional scoring-based measurements.

Different algorithms appear to perform differently under different scenarios. Previous studies have concluded that RF is a state-of-the-art algorithm for subtyping unilateral and bilateral PA and identifying patients with PA with essential hypertension.^6^^,^^8^^,^^21^ In our work, LightGBM was the best-performing algorithm among the 5 tested methods to identify *KCNJ5* mutations preoperatively. LightGBM is based on gradient boosting decision trees, which grow the trees sequentially instead of independently, and each tree corrects the errors of the previous tree. LightGBM further introduced an exclusive feature bundling and gradient-based 1-side sampling technique, which bundled the features without adding the loss and retained the samples that largely contributed to model performance.

### Application in real-world scenarios

Physicians are unlikely to diagnose a patient with a *KCNJ*5 mutation without advanced genomic sequencing of resected adrenal tissues, which requires invasive adrenalectomies or blind biopsies. The developed predictive models may increase the number of patients who undergo precisely recommended surgical treatment. The design of the condensed version is highly relevant for general hospitals because the selected parameters for this scenario are commonly available in general settings. Our findings enabled patient screening in general and tertiary hospitals, which is beneficial for making precise treatment options.

### Study limitations

Although we developed a more sophisticated classifier that decreases the burden of patient examination and data collection to facilitate the preoperative diagnosis of *KCNJ*5 mutations, several limitations need to be addressed. First, the model acts as a preoperative indication for uPAs harboring *KCNJ*5 mutations; however, this does not include the assessment of prognosis after surgery. To inspect the success of the surgery, further definition is required for the prognosis of the patients, follow-up of disease control, and quality of life of patients. Second, strong evidence has been proven for patients with uPA harboring *KCNJ5* mutation to opt for adrenalectomy or receive more aggressive treatment,^5^^,^^22^^,^^23^ genetic mutation information was often absent before adrenalectomy, which makes it challenging to encourage patients to undergo surgical treatments, especially those who respond well after mineralocorticoid receptor antagonist treatment. Finally, although 328 patients were considered as a small cohort, our work has an error between 0.05 and 0.10 under a 95% CI (requiring 102 to 408 participants) based on the sample size estimation method proposed by Riley et al^24^ and Skov et al.^25^ This indicates that the credibility of our study falls at a confidence level of 95%. Therefore, we considered the sample size of our study to be sufficient. The recruited patients came from different cities in Taiwan, including 2 medical centers, 3 affiliated hospitals, and 2 regional hospitals and was further validated by an international cohort. We did not conduct a randomized trial to recruit patients; instead, the patients were identified and approached during regular visits during the study period. The population represents the occurrence of cases observed in real practice, which is considered representative of the East Asian population.

## Conclusions

In this study, we developed machine learning models to identify the occurrence of *KCNJ*5 mutations in adrenal tissues of patients with uPA using baseline characteristics and routine blood/urine test results. We found that the full and condensed versions could accurately identify *KCNJ*5 mutations preoperatively. Our predictive model can potentially aid in providing better surgical options for patients with uPA who could benefit from ipsilateral adrenalectomies and guide postoperative following strategy.

Perspectives **COMPETENCY IN MEDICAL KNOWLEDGE:** Identification of uPA harboring *KCNJ*5 mutations requires adrenalectomy and sampling of the patient’s adrenal tumor tissues for Sanger sequencing, which is invasive and highly complex. This study is the first to apply a machine learning model to the preoperative identification of patients with uPA harboring *KCNJ*5 mutations, based only on basic demographic characteristics and laboratory test results.

**TRANSLATIONAL OUTLOOK:** The machine learning model was applied to the preoperative identification of patients with uPA harboring *KCNJ*5 mutations, and our work provides a personalized surgical recommendation to enable more confidence in advising patients to pursue surgical treatment. Therefore, although genomic mutation information is absent, the predictive model potentially broadens personalized assessment and increases the number of patients who undergo the recommended surgical treatment.

## Funding Support and Author Disclosures

This study was supported by the National Taiwan University Hospital (NTUH 100-N1776, 101-M1953, and 102-S2097), National Science Council in Taiwan (NSC 101-2314-B-002-132-MY3, NSC100-2314-B-002-119, and NSC 101-2314-B-002-085-MY3), and Ministry of Science and Technology in Taiwan (MOST 104-2314-B-002-125-MY3 and MOST 111-2314-B-075-011-MY3). The authors have reported that they have no relationships relevant to the contents of this paper to disclose.
