# Supplementary material for: Identifying KCNJ5 Mutation in Aldosterone-Producing Adenoma Patients With Baseline Characteristics Using Machine Learning Technology
Source: JACC Asia. 2023 Jun 13;3(4):664–75. doi: 10.1016/j.jacasi.2023.03.010 (PMC10442871; doi:10.1016/j.jacasi.2023.03.010)
Supplement: Supplemental Appendix [file mmc1.docx]

**SUPPLEMENTAL APPENDIX**

**Standard TAIPAI protocol and Aldosteronism Consensus in Taiwan**

**Patients were enrolled from the following hospitals^1^:**

This study included two medical centers (National Taiwan University Hospital (NTUH), Taipei, Taiwan; Taipei University Hospital, Taipei, Taiwan) and five regional hospitals (Cardinal Tien Hospital, New Taipei City, Taiwan; Taipei Tzu Chi Hospital, New Taipei City, Taiwan; Yun- Lin Branch of NTUH, Douliou City, Taiwan; Hsin-Chu Branch of NTUH, Hsin-Chu City, Taiwan; Zhongxing Branch of Taipei City Hospital, Taipei, Taiwan).

**Material and methods**

This study enrolled patients who were referred to the TAIPAI study group, including two tertiary medical centers, three affiliated hospitals, and two regional hospitals in different cities in Taiwan. Patients with other secondary hypertension, including, but not limited to, renovascular hypertension, Cushing’s syndrome, hyperthyroidism, and pheochromocytoma, were all excluded from this study. All antihypertensive medications were discontinued for at least 21 d before the PA screening tests. Doxazosin and/or diltiazem were administered to control the markedly high blood pressure when required. Patients who were confirmed to have family type I (FH-I)/ glucocorticoid-remediable aldosteronism were excluded from the analysis via a long-range polymerase chain reaction, as described previously.

All confirmed PA patients underwent a contrast-enhanced computed tomography (CT) scan of the abdomen with thin (3 mm) slices through the levels of the adrenal glands^2-12^

Ethical approval (approval number 200611031R) was obtained from the institutional review board of the National Taiwan University Hospital. Written informed consent for clinical data collection and research use was obtained from all participants before enrollment in the study. (<Http://doi.org/10.6084/m9.figshare.21730985>)

**Our standard protocol to identify primary aldosteronism (PA) and functional lateralization:**

Primary aldosteronism was diagnosed in patients with hypertension based on the following criteria.

***Confirmation***

Fulfillment of the following three conditions confirmed a diagnosis of PA ^10^:

(1) Autonomous excess aldosterone production evidenced by an aldosterone-renin ratio (ARR) > 35; (2) a TAIPAI score > 60%; (3) seated post-saline loading PAC > 16 ng/dL, or PAC/PRA > 35 (ng/dL)/(ng/mL/h) shown in a post-captopril/losartan test ^10^. (Abbreviations: PAC, plasma aldosterone concentration; PRA, plasma renin activity).

The probability of PA (TAIPAI score) was equal to:

= 1/(1 + e^–β^) ; where β = (PAC [ng/dl] × [0.063]) + PRA [ng/ml/h ] × [−0.205]) + ([ARR × 0.001] BMI [kg/m^2^] × [0.067]) + (Male × [−0.738] + SK [mmol/l] × [−1.512]) + (eGFR [ml/min/1.73 m^2^] × [0.017]) + ([propensity score] × [−0.539] + [1.851]) , (propensity score for eGFR<60 [ml/min/1.73 m^2^])

***Unilateral PA/clinical APA***

Unilateral PA/clinical (aldosterone-producing adenoma APA () was identified based on the following four criteria ^10^: (1) Confirmed PA; (2) adrenal adenoma or hyperplasia evidenced with a CT or MRI scan; and (3) lateralization of aldosterone secretion with adrenal vein sampling (AVS).

uPA is further confirmed after adrenalectomy:

(4) Pathologically proven positive CYP11B2-staining adenoma ^13^.

Bilateral PA was identified in the patients according to the following conditions: (1) bilateral diffuse enlargement of adrenal glands on a CT scan; (2) non-lateralization of aldosterone secretion by adrenal vein sampling or dexamethasone suppression NP-59 SPECT/CT; and (3) pathologically reported diffuse cell hyperplasia, namely, mAPM/mAPN at immunohistochemistry after adrenalectomy ^13^.

**Development of predictive models**

The model training process consists of three stages: data preparation, model training, and model validation, as illustrated in Fig. 2. During the data preparation stage, data imputation and feature normalization were performed. This work was implemented using Python v.3.7.6, and Scikit-learn v.0.22.1.

**Data preparation**

In the data preparation stage, we first explored the data, examined the type of missing values, performed data imputation, and investigated the difference between the original and imputed data to ensure its validity. In our study, patients were recruited from multiple sites, which inevitably included patients with incomplete data^1^. All cases had missing variables. Among the 87 parameters, patients with missing variables ranged from a maximum of 49 (56.32%) to a minimum of 3 (3.45%). Only 22 items (25.29%) were completed by all the patients. Therefore, omitting cases with incomplete data was impractical. Chi-square and Cramér's V tests ^2^ were performed to confirm the trend of the missing values. Our type of missing value falls into the category of missing at random (MAR), where the probability of non-response depends on the observed data and not on the values of the missing data. Therefore, data imputation was suggested^3^. In this study, missing values for features with numeric values (such as age, body weight, and height) were imputed using the mean values of all patients. Categorical features (such as smoking history) were imputed, with the most frequently appearing values ^4^. The correlation between the targeted variable (*KCNJ5* mutation) and each feature was tested using Pearson’s correlation test to verify whether dominant variables were included.

Data imputation is intended to preserve the relationships between variables and produce valid analytic results rather than to estimate, recover, or replace missing values^5^. We investigated the correlation between *KCNJ5* mutation and each parameter before and after imputation to ensure that the variable relationships remained unchanged (shown in Table 2 and Supplemental Table 4). The results show that after imputation, the correlation changes varied by less than 0.1, which is considered to preserve the original relationship. The targeted variable after imputation had no higher correlation than ±0.4; the other variables, which were considered insignificant, did not potentially affect the prediction result as a dominant indicator. Therefore, we concluded that data imputation was an appropriate approach and preserved the original distribution of the patient data.

Finally, the data were normalized and encoded into dummy variables. Owing to the nature of the exclusive characteristics of individual measurements, normalization is essential for scaling different features into a specific interval. This process enables the model to consider each feature equally, thereby achieving better accuracy and minimizing errors ^6, 7^. Here, we used the min-max normalization technique and scaled each continuous feature to a range of –1 to 1. Categorical features were encoded in a one-hot encoding form^8^, which converts a categorical variable that has $n$ values into $n$ variables. After normalization, the features were concatenated into a vector, with each row representing an individual patient.

**Model training**

We tested multiple machine learning algorithms, including logistic regression (LR) ^9^, gaussian radial basis function kernelled support vector machine (kernelled SVM), random forest (RF), eXtreme gradient boosting (XGBoost), and light gradient boosting machine (LightGBM), to find the classifier with the best performance. The proposed model was designed as a binary classifier that predicts whether the patient has a *KCNJ5* mutation or a wild-type [WT] *KCNJ5* carrier. In the model training stage, the data were first stratified into training and testing datasets at an 80:20 ratio, ensuring that each dataset contained approximately the same proportions of the two types of prediction labels (i.e., the number of patients with *KCNJ5* mutations and without mutations were balanced). The data were split based on random assignments, indicating that the training and testing datasets were homogeneous. Five-fold cross-validation was performed. Earlier studies have demonstrated that utilizing multiple splits into folds for repeating cross-validation leads to more dependable performance estimation ^17, 18^. The training dataset was divided into five subsets, where each subset was rotated as a validation subset and the others were used as training subsets. Our research entails a limited sample size, therfore, we prevent overfitting by carefully monitoring the performance during both training and testing phases. As a result, we have chosen to partition the dataset into training and testing subsets, and then conduct 5-fold cross-validation on the training subset to improve the accuracy of the estimation. By using a separate testing subset, we have ensured that the performance validation is authentic and that data leakage problems are avoided.

This research tested several classifiers, including Logistic Regression (LR), Gaussian Radial Basis Function Kernelled Support Vector Machine (kernelled SVM), Random Forest (RF), extreme Gradient Boosting (XGBoost), and Light Gradient Boosting Machine (LightGBM). The hyperparameter settings for LR were the default settings, with the maximum number of iterations taken for the classifier to converge to 100000, and the regularization parameter $C$ was set to 6. The gamma parameter was set to “auto,” which indicates using 1/$F_{n}$ as kernel coefficient, where $F_{n}$denotes as the number of features that were included; The number of trees set for RF was 100, having the maximum depth of the tree set to 10; For XGBoost, the number of trees was set to 800, with the maximum depth of the tree as 60, using 0.05 as learning rate, and the minimum sum of instance weight needed in a child was set to 20; In LightGBM, the max number of leaves in one tree is set to 4, the learning rate was set to 0.05, using 100 epoch to reach converge. The detailed ablation experiment of the hyperparameters is presented in Supplemental Table 8.

**Model validation**

Finally, in the model validation stage, the model was evaluated based on the testing dataset, which was isolated at the beginning of the training process and considered the unseen data. The evaluation metrics included the receiver operating characteristic (ROC) curve, the area under the curve (AUC) for the ROC, accuracy, sensitivity, specificity, precision, and the F1 score. The AUC refers to the true distribution of positive and negative instances ^10^. Accuracy refers to the ratio of correctly classified samples to the overall number of samples; sensitivity, also known as recall, represents the ability to correctly classify patients with positive results; specificity indicates correctly classifying patients with negative results; precision indicates that the proportion of correct positive identifications; and the F1 score represents the harmonic mean of precision and sensitivity ^11^. The calculation equation of the metrics were listed as equation (1) – (5), where true positive (TP) indicates a positive result that is truly positive, true negative (TN) indicates a negative result that is truly negative, false positive (FP) indicates a positive result that is actually negative, and false negative (FN) is when the negative result is actually positive. The robustness of the model was based on its general performance in all aspects. Therefore, the evaluation metrics were averaged, and an average score was calculated to compare the approaches. To estimate the performance variation of the prediction model, we used a non-parametric bootstrap approach to identify the 95% confidence interval (CI) ^12^, indicating the boundaries of possible values.

$Accuracy = \left( TP+TN \right) / \left( TP+FP+FN+TN \right)$ (1)

$Sensitivity= TP / \left( TP+FN \right)$ (2)

$Specificity = TN / \left( TN+FP \right)$ (3)

$Precision = TP / \left( TP+FP \right)$ (4)

$F1 = 2\times\left( \mathrm{Prescision}\times S\mathrm{ensitivity} \right) / \left( Prescision+Sensitivity \right)$ (5)

**Model design of the condensed version**

When identifying distinguishable variables that can separate patients with uPA harboring *KCNJ*5 mutations from those with WT-*KCNJ5* carriers, a straightforward approach is to perform a statistical t-test and determine which variable appears to be statistically significant. Therefore, we used the statistical results to indicate sensible variables. A common approach in machine learning is to rank the feature importance of variables, which indicates the contribution of features during classification. Here, we used feature importance ranking as another parameter-condensation method. The statistical and feature importance versions were compared and discussed to determine the best condensing approach ^13^.

**Model training based on character-specific variables and external validation**

Further, owing to the characteristics of uPA, we additionally trained models to investigate character-specific impacts. The prevalence of hypokalemia has been reported to differ among PA subtypes ^14, 15^ and a large proportion of patients with PA present with normokalemia. Therefore, we further trained an additional model to investigate the model performance when excluding patients with hypokalemia. It has been observed that *KCNJ5* mutations occur more frequently in Western women ^16^. However, such a gender dimorphism has not been reported in East Asian populations [8]. To further assess the impact of sex differences among uPA patients, we additionally trained the prediction model based on male and female patients. The character-specific models were trained using the full examination version, excluding hypokalemia or sex. We intend to exhibit the robustness and generalization of our model and further perform an external validation based on data from the National University of Malaysia (UKM) Medical Center PA cohort. The data received comprised 14 parameters owing to local practice limitations (Supplemental Table 4). Therefore, we additionally trained an extremely condensed version based on the Taiwan data to comply with the Malaysian dataset. External validation was performed using the Malaysian dataset. The baseline characteristics of the Malaysian data were shown in Supplemental Table 7. The Malaysian data were imputed and normalized using the same process as Taiwan dataset, but were not involved in the training process. The reported results demonstrated the exclusive validation of 27 patients.

**Supplemental Table 1.** No statistically significant characteristics were observed in patients with primary aldosteronism with or without KCNJ5 mutations.

| Predictors | All  N=328 | *WT- KCNJ5*  N= 158 | *KCNJ*5 mutations  N= 170 | p-value |
| --- | --- | --- | --- | --- |
| Baseline Characteristics | | | |  |
| Gender (Male, n) | 154 (47.0) | 79 (50%) | 75 (44.1%) | 0.283 |
| Height (cm) | 163.58 (8.8) | 163.82 (8.8) | 165.34 (8.9) | 0.628 |
| Body Weight (kg) | 68.54 (15.0) | 69.98 (15.3)) | 67.23 (14.61) | 0.097 |
| BMI (kg/m^2^) | 25.39 (4.4) | 25.88 (4.2) | 25.08 (4.2) | 0.088 |
| BSA | 1.74 (0.21) | 1.75 (0.21) | 1.72 (0.21) | 0.180 |
| Buttocks (cm) | 95.77 (9.7) | 96.62 (9.7) | 95.10 (9.6) | 0.214 |
| SBP (mmHg) | 153.97 (22.3) | 154.52 (23.0) | 153.76 (21.5) | 0.757 |
| DBP (mmHg) | 91.72 (14.8) | 91.40 (13.6) | 92.36 (15.1) | 0.553 |
| Heart rate (beat per minute) | 73.23 (13.4) | 73.85 (14.6) | 72.59 (12.2) | 0.401 |
| Comorbidities | | | |  |
| Smoking (n) | 51 (15.5) | 28 (17.7) | 23 (13.5) | 0.274 |
| CAD (n) | 25 (7.6) | 15 (9.5) | 10 (5.9) | 0.218 |
| COPD (n) | 0 (0) | 0 (0) | 0 (0) | -- |
| MI (n) | 0 (0) | 0 (0) | 0 (0) | -- |
| CVA (n) | 21 (6.4) | 11 (7) | 10 (5.9) | 0.689 |
| Renal stone (n) | 18 (5.5) | 11 (7) | 7 (4.1) | 0.258 |
| Hyperlipidemia (n) | 76 (23.2) | 44 (27.8) | 32 (18.8) | 0.052 |
| Diabetics (n) | 52 (15.9) | 30 (19.0) | 22 (12.9) | 0.133 |
| Family hypertension (n) | 209 (63.7) | 101 (63.9) | 108 (63.5) | 0.931 |
| Congestive heart failure (n) | 2 (0.6) | 2 (1.3) | 0 (0) | 0.141 |
| Atrial fibrillation (n) | 10 (3.0) | 4 (2.5) | 6 (3.5) | 0.660 |
| HBV (n) | 42 (12.8) | 24 (15.2) | 18 (10.6) | 0.212 |
| HCV (n) | 3 (0.9) | 2 (1.3) | 1 (0.6) | 0.519 |
| Liver cirrhosis (n) | 1 (0.3) | 0 (0) | 1 (0.6) | 0.334 |
| Chronic glomerulonephritis (n) | 0 (0) | 0 (0) | 0 (0) | -- |
| Hyperthyroidism (n) | 3 (0.9) | 2 (1.3) | 1 (0.6) | 0.519 |
| Hypothyroidism (n) | 4 (1.2) | 1 (0.6) | 3 (1.8) | 0.351 |
| Hypertension drug (n) | 2.48 (1.4) | 2.42 (1.5) | 2.54 (1.3) | 0.422 |
| Laboratory Data | | | |  |
| Plasma Renin Activity (PRA) (ng/ml/hr) | 5.84 (90.1) | 1.17 (4.1) | 0.53 (1.1) | 0.055 |
| Aldosterone Over Plasma (ARR) | 1100.24 (2409.3) | 992.47 (2140.5) | 1208.13 (2644.6) | 0.422 |
| Serum Cl^-^ (mmol/L) | 102.69 (5.03) | 102.93 (5.05) | 102.46 (5.03) | 0.535 |
| Serum Mg^2+^ (mmol/L) | 0.97 (0.34) | 0.98 (0.36) | 0.93 (0.27) | 0.382 |
| Serum P^3-^ (mg/dL) | 3.38 (0.79) | 3.42 (0.60) | 3.33 (0.93) | 0.401 |
| HOMA (Glucose*Insulin) / 405) | 120.57 (527.8) | 87.90 (452.2) | 152.56 (592.3) | 0.297 |
| Serum HbA_1_c (%) | 6.14 (1.23) | 6.21 (1.09) | 6.02 (1.47) | 0.593 |
| Blood osmolarity (mOsm/Kg) | 290.14 (5.52) | 290.15 (5.35) | 290.06 (5.65) | 0.907 |
| Serum HDL (mg/dL) | 47.28 (13.29) | 46.54 (13.71) | 48.03 (12.92) | 0.356 |
| Serum LDL (mg/dL) | 110.13 (30.4) | 113.40 (32.46) | 106.97 (28.08) | 0.094 |
| Serum cortisol 8 am (μg/dL) | 12.24 (6.41) | 11.84 (6.67) | 12.72 (6.08) | 0.308 |
| Serum cortisol 4 pm (μg/dL) | 8.60 (4.91) | 8.78 (5.33) | 8.42 (4.51) | 0.744 |
| Serum ACTH 4 pm (pg/mL) | 14.81 (9.51) | 12.95 (7.84) | 16.63 (10.67) | 0.077 |
| Serum DHEAS (μmol/L) | 7.32 (33.0) | 3.51 (5.20) | 11.89 (48.71) | 0.308 |
| Serum cystatin (mg/L) | 0.84 (0.33) | 0.86 (0.37) | 0.82 (0.29) | 0.448 |
| Serum CRP (mg/dL) | 1.11 (2.43) | 1.43 (2.95) | 0.75 (1.61) | 0.159 |
| Serum Hb (g/dL) | 13.41 (1.61) | 13.6 (1.63) | 13.3 (1.60) | 0.105 |
| Serum platelet (k/μL) | 236.91 (62.81) | 235.34 (66.4) | 238.26 (60.07) | 0.728 |
| 24 hours urine amount (cc/24hr) | 2290.71 (731.74) | 2264.34 (751.76) | 2311.32 (720.35) | 0.650 |
| Urine UA (mg/dL) | 32.78 (18.41) | 34.55 (18.48) | 31.20 (18.39) | 0.220 |
| Urine K^+^ (mmol/L) | 28.12 (16.00) | 26.77 (14.26) | 29.05 (17.13) | 0.254 |
| Urine Ca^2+^ (mmol/L) | 2.64 (2.01) | 2.71 (2.03) | 2.59 (2.01) | 0.676 |
| Urine Mg^2+^ (mmol/L) | 1.62 (0.92) | 1.76 (1.24) | 1.51 (0.58) | 0.294 |
| Urine P^3-^ (mg/dL) | 35.84 (20.53) | 37.14 (19.38) | 34.68 (21.54) | 0.390 |
| Urine microalbumin (mg/L) | 45.10 (147.54) | 61.12 (199.86) | 30.69 (71.14) | 0.139 |
| Urine Microalbumin Over Creatinine (UACR) | 3.33 (2.93) | 3.37 (2.92) | 3.30 (2.97) | 0.879 |
| 24 hours amount urine VMA (mg/24hr) | 2298.85 (767.46) | 2248.30 (801.82) | 2363.44 (720.44) | 0.288 |
| Urine VMA (mg) | 4.01 (1.81) | 4.06 (2.02) | 3.94 (1.51) | 0.596 |
| Urine cortisol (μg/dL) | 9.54 (16.86) | 9.28 (16.47) | 9.85 (17.56) | 0.886 |
| 24 hours urine catecholamine (μg/24hr) | 2270.66 (761.19) | 2229.38 (778.97) | 2317.28 (742.42) | 0.440 |
| Urine dopamine (μg/dL) | 218.47 (99.75) | 215.02 (95.33) | 222.34 (104.87) | 0.610 |
| Urine epinephrine (μg/dL) | 10.67 (30.78) | 7.18 (6.38) | 13.66 (41.47) | 0.286 |
| Urine norepinephrine (μg/dL) | 42,30 (23.23) | 40.21 (21.68) | 44.61 (24.77) | 0.189 |

ACTH, adrenocorticotropic hormone; BMI, body mass index; BSA, body surface area; Ca^2+^+, calcium; CAD, coronary artery disease; Cl^-^, chloride; COPD, chronic obstructive pulmonary disease; CRP, C-reactive protein; CVA, cerebrovascular accident; DBP, diastolic blood pressure; DHEAS, dehydroepiandrosterone sulfate; HbA_1_c, hemoglobin A1c; HBV, hepatitis B virus; HCV, hepatitis C virus; HDL, high-density lipoprotein; HOMA, homeostatic model assessment; K^+^, potassium; LDL, low-density lipoprotein; Mg^2+^, magnesium; MI, myocardial infarction; P^3-^, phosphate; SBP, systolic blood pressure; VMA, vanillylmandelic acid; WT, wild-type

All continuous parameters were presented as mean (standard deviation)

All categorical parameters were presented as numbers (percentages)

**Supplemental Table 2.** Correlation between the target variable (KCNJ5 mutation) and each variable using the Pearson correlation test and the number of missing values for each variable.

| **Variables** | **Correlation between KCNJ5 mutation and each parameter** | | | **Number of missing values for each parameter** | **(%)** |
| --- | --- | --- | --- | --- | --- |
|  | **Before imputation** | **After imputation** | **Variance** |  |  |
| Height (cm) | -0.029 | -0.029 | 0.000 | 2 | 0.610 |
| Body weight (Kg) | -0.092 | -0.092 | 0.000 | 2 | 0.610 |
| BSA | -0.079 | -0.079 | 0.000 | 2 | 0.610 |
| Waistline (cm) | -0.228 | -0.200 | 0.028 | 74 | 22.561 |
| Buttocks (cm) | -0.087 | -0.077 | 0.010 | 73 | 22.256 |
| Heart rate (beat per minute) | -0.047 | -0.047 | 0.000 | 5 | 1.524 |
| Age (y/o) | -0.276 | -0.275 | 0.001 | 2 | 0.610 |
| BMI (kg/m^2^) | -0.099 | -0.099 | 0.000 | 2 | 0.610 |
| Latency of HTN (year) median [IQR] | -0.150 | -0.146 | 0.004 | 4 | 1.220 |
| SBP (mmHg) | -0.018 | -0.018 | 0.000 | 2 | 0.610 |
| DBP (mmHg) | 0.033 | 0.033 | 0.000 | 2 | 0.610 |
| Smoking (n) | -0.060 | -0.060 | 0.000 | 0 | 0.000 |
| Atrial fibrillation (n) | 0.028 | 0.028 | 0.000 | 0 | 0.000 |
| Myocardial Infarction (n) | -- | -- | -- | 0 | 0.000 |
| Congestive heart failure (n) | -0.082 | -0.082 | 0.000 | 0 | 0.000 |
| COPD (n) | -- | -- | -- | 0 | 0.000 |
| HBV (n) | -0.071 | -0.071 | 0.000 | 0 | 0.000 |
| HCV (n) | -0.036 | -0.036 | 0.000 | 0 | 0.000 |
| Liver Cirrhosis (n) | 0.053 | 0.053 | 0.000 | 0 | 0.000 |
| Chronic glomerulonephritis (n) | -- | -- | -- | 0 | 0.000 |
| Gender (M) (n) | 0.059 | 0.059 | 0.000 | 0 | 0.000 |
| Family hypertension (n) | 0.000 | -0.001 | 0.000 | 2 | 0.610 |
| Hypertension drug (n) | 0.042 | 0.042 | 0.042 | 0 | 0.000 |
| Hypertension (n) | 0.127 | 0.127 | 0.000 | 0 | 0.000 |
| Diabetics (n) | -0.085 | -0.085 | 0.000 | 0 | 0.000 |
| Hyperlipidemia (n) | -0.110 | -0.110 | 0.000 | 0 | 0.000 |
| Hyperthyroidism (n) | 0.051 | 0.051 | 0.000 | 0 | 0.000 |
| Hypothyroidism (n) | -0.005 | -0.005 | 0.000 | 0 | 0.000 |
| Hypokalemia (n) | 0.232 | 0.232 | 0.000 | 0 | 0.000 |
| CAD (n) | -0.070 | -0.070 | 0.000 | 0 | 0.000 |
| CVA (n) | -0.024 | -0.024 | 0.000 | 0 | 0.000 |
| Renal stone (n) | -0.064 | -0.064 | 0.000 | 0 | 0.000 |
| Serum BUN (mg/dL) | -0.155 | -0.144 | 0.011 | 46 | 14.024 |
| Serum creatinine (mg/dL) | -0.116 | -0.116 | 0.000 | 2 | 0.610 |
| Serum UA (mg/dL) | -0.167 | -0.152 | 0.014 | 54 | 16.463 |
| Serum Ca^2+^ (mmol/L) | -0.024 | -0.023 | 0.002 | 39 | 11.890 |
| Serum Cl^-^ (mmol/L) | -0.047 | -0.035 | 0.013 | 153 | 46.646 |
| Serum Mg^2+^ (mmol/L) | -0.046 | -0.024 | 0.022 | 238 | 72.561 |
| Serum P^3-^ (mg/dL) | -0.053 | -0.043 | 0.010 | 109 | 33.232 |
| Serum glucose (mg/dL) | -0.123 | -0.119 | 0.004 | 21 | 6.402 |
| Serum insulin (μIU/ml) | -0.161 | -0.124 | 0.038 | 125 | 38.110 |
| HOMA (Glucose*Insulin) / 405) | 0.060 | 0.057 | 0.003 | 36 | 10.976 |
| Serum HbA_1_c () | -0.072 | -0.029 | 0.043 | 270 | 82.317 |
| Blood osmolarity (mOsm/Kg) | -0.010 | -0.008 | 0.002 | 100 | 30.488 |
| Serum cholesterol (mg/dL) | -0.115 | -0.106 | 0.009 | 47 | 14.329 |
| Serum triglyceride (mg/dL) | -0.266 | -0.250 | 0.015 | 37 | 11.280 |
| Serum HDL (mg/dL) | 0.056 | 0.052 | 0.005 | 52 | 15.854 |
| Serum LDL (mg/dL) | -0.105 | -0.092 | 0.013 | 75 | 22.866 |
| pH | 0.219 | 0.167 | 0.052 | 136 | 41.463 |
| Serum HCO_3_^-^ (mmol/L) | 0.324 | 0.247 | 0.078 | 137 | 41.768 |
| Serum cortisol 8 am (μg/dL) | 0.066 | 0.054 | 0.012 | 106 | 32.317 |
| Serum ACTH 8 am (pg/dL) | 0.151 | 0.125 | 0.027 | 104 | 31.707 |
| Serum cortisol 4 pm (μg/dL) | -0.036 | -0.018 | 0.018 | 245 | 74.695 |
| Serum ACTH 4 pm (pg/dL) | 0.195 | 0.098 | 0.097 | 245 | 74.695 |
| Serum DHEAS (μmol/L) | 0.127 | 0.057 | 0.070 | 260 | 79.268 |
| Serum cystatin (mg/L) | -0.055 | -0.044 | 0.011 | 118 | 35.976 |
| Serum CRP (mg/dL) | -0.147 | -0.082 | 0.065 | 223 | 67.988 |
| Serum i-PTH (pg/mL) | 0.185 | 0.125 | 0.060 | 179 | 54.573 |
| Lowest serum K^+^ (mmol/L) | -0.276 | -0.276 | 0.000 | 0 | 0.000 |
| Serum hemoglobin | -0.109 | -0.090 | 0.018 | 100 | 30.488 |
| Serum platelet (k/μL) | 0.023 | 0.019 | 0.004 | 99 | 30.183 |
| Serum Na^+^(mmol/L) | 0.272 | 0.270 | 0.003 | 6 | 1.829 |
| Serum K^+^ (mmol/L) | -0.439 | -0.439 | 0.000 | 0 | 0.000 |
| Serum aldosterone (ng/dL) | 0.140 | 0.140 | 0.000 | 2 | 0.610 |
| Plasma Renin Activity (PRA) (ng/ml/hr) | -0.126 | -0.126 | 0.000 | 2 | 0.610 |
| Aldosterone Over Plasma (ARR) | 0.057 | 0.057 | 0.000 | 2 | 0.610 |
| Urine aldosterone (μg/24hrs) | 0.191 | 0.149 | 0.041 | 126 | 38.415 |
| Urine creatinine (mg/dL) | -0.145 | -0.126 | 0.019 | 80 | 24.390 |
| Urine UA (mg/dL) | -0.093 | -0.070 | 0.023 | 141 | 42.988 |
| Urine K^+^ (mmol/L) | 0.078 | 0.069 | 0.009 | 72 | 21.951 |
| Urine Na^+^ (mmol/L) | -0.208 | -0.177 | 0.031 | 89 | 27.134 |
| Urine Ca^2+^ (mmol/L) | -0.026 | -0.021 | 0.005 | 105 | 32.012 |
| Urine Mg^2+^ (mmol/L) | -0.137 | -0.058 | 0.078 | 267 | 81.402 |
| Urine P^3-^ (mg/dL) | -0.061 | -0.048 | 0.012 | 118 | 35.976 |
| Urine Cl^-^ (mmol/L) | -0.148 | -0.108 | 0.040 | 153 | 46.646 |
| Urine osmolarity (mOsm/Kg) | -0.139 | -0.115 | 0.024 | 102 | 31.098 |
| Urine microalbumin (mg/L) | -0.104 | -0.083 | 0.021 | 101 | 30.793 |
| Urine Microalbumin Over Creatinine (UACR) | -0.080 | -0.063 | 0.017 | 106 | 32.317 |
| TTKG | 0.299 | 0.236 | 0.063 | 123 | 37.500 |
| 24 hours amount urine VMA (mg/24hr) | 0.075 | 0.059 | 0.016 | 123 | 37.500 |
| Urine VMA (mg) | -0.035 | -0.029 | 0.006 | 99 | 30.183 |
| Urine cortisol (μg/dL) | 0.017 | 0.008 | 0.009 | 248 | 75.610 |
| 24 hours urine catecholamine (μg/24hr) | 0.058 | 0.043 | 0.015 | 147 | 44.817 |
| Urine dopamine (μg/dL) | 0.037 | 0.028 | 0.008 | 133 | 40.549 |
| Urine epinephrine (μg/dL) | 0.106 | 0.059 | 0.046 | 134 | 40.854 |
| Urine norepinephrine (μg/dL) | 0.095 | 0.073 | 0.022 | 134 | 40.854 |

ACTH, adrenocorticotropic hormone; BMI, body mass index; BSA, body surface area; BUN, blood urea nitrogen; Ca^2+^, calcium; CAD, coronary artery disease; Cl^-^, chloride; COPD, chronic obstructive pulmonary disease; CRP, C-reactive protein; CVA, cerebrovascular accident; DBP, diastolic blood pressure; DHEAS, dehydroepiandrosterone sulfate; HbA_1_c, hemoglobin A1c; HBV, hepatitis B virus; HCV, hepatitis C virus; HDL, high-density lipoprotein; HOMA, homeostatic model assessment; K^+^, potassium; LDL, low-density lipoprotein; Mg^2+^, magnesium; P^3-^, phosphate; SBP, systolic blood pressure; VMA, vanillylmandelic acid; ACTH, adrenocorticotropic hormone; HCO_3_^-^, bicarbonate; HTN, hypertension; i-PTH, intact parathyroid hormone; IQR, interquartile range; K^+^, potassium; Na^+^, sodium; TTKG, transtubular potassium gradient

**Supplemental Table 3.** Summary of chi-square and Cramer’s V tests to validate the distribution of missing and observed values between each parameter pairwise.

|  | Number of pairwise variables (%) | Mean Cramers’ V |
| --- | --- | --- |
| Total | 4160 (100.00) | 0.210 |
| Indepandent (p > 0.05) | 1746 (41.97) | 0.048 |
| Depandant (p < 0.05) | 2414 (58.03) | 0.327 |

**Supplemental Table 4. Different approaches to full or condense required parameters**

| **a. Full features (n=87)** |
| --- |
| **Baseline Characteristics (n=12)**  Gender, Height, Waistline, Age, Body Weight, BMI, BSA, Buttocks, SBP, DBP, Heart rate,  **Comorbidities (n=21)**  Smoking, CAD, COPD, MI, CVA, Renal stone, hyperlipidemia, diabetics, Family hypertension, Congestive heart failure, Atrial fibrillation, HBV, HCV, Liver cirrhosis, Chronic glomerulonephritis, Hyperthyroidism, Hypothyroidism, taking a Hypertension drug, Hypokalemia, Hypertension, Hypertension duration,  **Laboratory Data (serum or plasma, n=33)**  PRA, ARR, Serum Cl^-^, Serum Mg^2+^, HOMA, Serum HbA_1_c, Blood osmolarity, Serum HDL, 8 am Serum cortisol, 4 pm Serum cortisol, 4 pm Serum ACTH, Serum DHEAS, Serum cystatin, Serum CRP, Serum Hb, Serum platelet, Serum P^3-^, Lowest serum potassium level, Serum potassium level, Serum aldosteronism, Serum BUN, Serum creatinine, Serum Na^+^, Serum calcium, Serum urine acid, Serum glucose, Serum insulin, Serum triglyceride, Serum cholesterol, Blood pH, Serum bicarbonate (HCO_3_^-^), Serum ACTH 8 AM, i-PTH  **Laboratory Data (urine, n=21)**  Urine aldosterone, Urine creatinine, Urine Na^+^, Urine osmolality, Urine Cl^-^, trans-tubular potassium gradient (TTKG), 24 hours urine amount, Urine UA, Urine K^+^, Urine Ca^2+^, Urine Mg^2+^, Urine P^3-^, Urine microalbumin, Urine Microalbumin Over Creatinine (UACR), 24 hours amount urine VMA, Urine VMA, Urine cortisol, 24 hours urine catecholamine, Urine dopamine, Urine epinephrine, Urine norepinephrine |
| **b. Statistically significant features (n=27)** |
| **Baseline Characteristics (n=2)**  Waistline, Age  **Comorbidities (n=3)**  Hypokalemia, Hypertension, hypertension duration,  **Laboratory Data (serum or plasma, n=16)**  Lowest serum potassium level, Serum potassium level, Serum aldosteronism, Serum BUN, Serum creatinine, Serum Na^+^, Serum calcium, Serum urine acid, Serum glucose, Serum insulin, Serum triglyceride, Serum cholesterol, Blood pH, Serum bicarbonate (HCO_3_^-^), 8 am Serum ACTH, i-PTH  **Laboratory Data (urine, n=6)**  Urine aldosterone, Urine creatinine, Urine Na^+^, Urine osmolality, Urine Cl^-^, trans-tubular potassium gradient (TTKG) |
| **c. Top 27 feature importance ranking** |
| **Baseline Characteristics (n=6)**  Waistline, Age, BMI, Body weight, DBP, SBP  **Comorbidities (n=1)**  Hypokalemia  **Laboratory Data (serum or plasma, n=12)**  Lowest serum potassium level, Serum potassium level, Serum Na^+^, Serum calcium, Serum insulin, Serum triglyceride, Serum bicarbonate (HCO_3_^-^), 8 am Serum ACTH, 8 am Serum cortisol, homeostatic model assessment (HOMA), Serum HDL, ARR  **Laboratory Data (urine, n=8)**  Urine aldosterone, Urine Na^+^, Urine K^+^, Urine Cl^-^, Urine Ca^2+^, trans-tubular potassium gradient (TTKG), Urine microalbumin, Urine Microalbumin over creatinine (UACR) |
| **d. Extremely condensed version** |
| Gender, Age, BMI, SBP, DBP, Heart Rate, Hypertension duration, Hypokalemia, Serum Na, Serum K, Serum Aldosterone, PRA, Serum Creatinine, Serum cortisol 8 am |

ACTH, adrenocorticotropic hormone; ARR, Aldosterone Over Plasma; BMI, body mass index; BSA, body surface area; BUN, blood urea nitrogen; Ca^2+^, calcium; CAD, coronary artery disease; Cl^-^, chloride; CRP, C reactive protein; COPD, chronic obstructive pulmonary disease; CVA, cerebrovascular accident; DBP, diastolic blood pressure; DHEAS, dehydroepiandrosterone sulfate; HbA_1_c, hemoglobin A1c; HBV, hepatitis B virus; HCV, hepatitis C virus; HCO_3_^-^, bicarbonate; HDL, high-density lipoprotein; HOMA, homeostatic model assessment; HTN, hypertension; i-PTH, intact parathyroid hormone; K^+^, potassium; Na^+^, sodium; LDL, low-density lipoprotein; Mg^2+^, magnesium; MI, myocardial infarction; P^3-^, phosphate; PRA, Plasma Renin Activity; SBP, systolic blood pressure; TTKG, trans-tubular potassium gradient; UA, uric acid; VMA, vanillylmandelic acid; WT, wild type.

In the condensed required parameters, 14 features overlapped, including waistline, age, hypokalemia, lowest serum potassium level, serum potassium level, serum calcium, serum insulin, serum triglyceride, serum bicarbonate (HCO3-), 8 am serum ACTH, urine aldosterone, urine Na^+^, urine Cl^-^, and trans-tubular potassium gradient (TTKG).

**Supplemental Table 5.** Top 27 ranked important features of other tested algorithms

| **Variables** | Logistic Regression | | Random Forest | | Kernelled SVM | | XGBoost | |
| --- | --- | --- | --- | --- | --- | --- | --- | --- |
| 1 | Serum sodium | 1.192 | Lowest serum potassium | 0.075 | Serum potassium | 0.070 | Lowest serum potassium | 0.311 |
| 2 | Hypokalemia | 1.116 | Serum potassium | 0.056 | Age | 0.035 | Serum potassium | 0.152 |
| 3 | Urine potassium | 0.758 | Age | 0.038 | Hypokalemia | 0.020 | Serum triglyceride | 0.053 |
| 4 | Hypertension | 0.696 | ARR | 0.030 | Waistline | 0.012 | Age | 0.052 |
| 5 | Serum bicarbonate | 0.592 | Serum Triglyceride | 0.029 | Lowest serum potassium | 0.010 | ARR | 0.052 |
| 6 | Urine aldosterone | 0.539 | Serum 8 am ACTH | 0.026 | Serum sodium | 0.008 | Serum calcium | 0.050 |
| 7 | Hip circumference | 0.479 | Serum calcium | 0.023 | Urine sodium | 0.007 | Hypertension duration | 0.049 |
| 8 | Urine Norepinephrine | 0.449 | Serum bicarbonate | 0.021 | TTKG | 0.006 | BMI | 0.048 |
| 9 | TTKG | 0.413 | TTKG | 0.019 | Urine potassium | 0.005 | Serum 8 am ACTH | 0.039 |
| 10 | Serum 4 pm ACTH | 0.412 | Waistline | 0.019 | Serum bicarbonate | 0.004 | Serum sodium | 0.027 |
| 11 | Urine phosphate | 0.410 | Serum aldosterone | 0.018 | Without Hypertension drug | 0.004 | Serum aldosterone | 0.016 |
| 12 | Urine cortisol | 0.383 | Serum sodium | 0.017 | Serum triglyceride | 0.004 | Serum uric acid | 0.015 |
| 13 | Serum chloride | 0.377 | Serum uric acid | 0.017 | Urine creatinine | 0.004 | Waistline | 0.011 |
| 14 | Hemoglobin A1c | 0.357 | BMI | 0.017 | Serum LDL | 0.003 | Body weight | 0.010 |
| 15 | Serum 8 am cortisol | 0.357 | Blood urea nitrogen | 0.017 | Blood pH | 0.003 | Serum HDL | 0.010 |
| 16 | Atrial fibrillation | 0.357 | Plasma renin activity | 0.016 | Serum aldosterone | 0.003 | Systolic blood pressure | 0.010 |
| 17 | 4 kinds of Hypertension drug | 0.355 | Urine sodium | 0.016 | Hypertension Duration | 0.003 | Heart rate | 0.009 |
| 18 | HOMA | 0.326 | Blood pH | 0.016 | Plasma renin activity | 0.002 | Serum glucose | 0.009 |
| 19 | DHEA_SO4 | 0.320 | Serum glucose | 0.015 | Hypertension | 0.002 | Urine sodium | 0.008 |
| 20 | Serum Osmolarity | 0.313 | 24 hours urine amount VMA | 0.015 | HOMA | 0.002 | Blood urea nitrogen | 0.008 |
| 21 | Aldosterone | 0.305 | Urine aldosterone | 0.015 | Serum 8 am Cortisol | 0.002 | Urine creatinine | 0.008 |
| 22 | Serum 8 am ACTH | 0.264 | Heart rate | 0.015 | 1 kind of Hypertension drug | 0.002 | UACR | 0.006 |
| 23 | EpinephrineU | 0.238 | Serum 8 am cortisol | 0.015 | Serum Insulin | 0.001 | HOMA | 0.006 |
| 24 | Urine Osmolarity | 0.208 | Systolic blood pressure | 0.014 | Serum phosphate | 0.001 | Serum creatinine | 0.006 |
| 25 | Blood urea nitrogen | 0.200 | Serum cholesterol | 0.014 | Serum Creatinine | 0.001 | Urine microalbumin | 0.005 |
| 26 | 3 kinds of Hypertension drug | 0.186 | Hypertension duration | 0.014 | Hepatitis B virus | 0.001 | Serum cholesterol | 0.005 |
| 27 | Blood pH | 0.183 | i-PTH | 0.014 | Urine norepinephrine | 0.001 | Urine potassium | 0.005 |

Owing to the natural differences between the algorithms, the importance was calculated based on the attributes of the algorithms to determine the most effective variables. LR, coefficients of the variable; RF, internal variable importance; Kernelled SVM, permutation importance; XGBoost, internal feature importance.

ACTH, adrenocorticotropic hormone; ARR, Aldosterone Over Plasma; BMI, body mass index; DHEAS, dehydroepiandrosterone sulfate; HOMA, homeostatic model assessment; i-PTH, intact parathyroid hormone; TTKG, trans-tubular potassium gradient; UACR, urine Microalbumin over creatinine

**Supplemental Table 6.** Additional trained model based on a specific population compared with the full examination version.

|  | Full examination version | Condensed Version | No Hypokalemia version | Male Version | Female Version | Extremely condensed version | External validation based on Malaysian data |
| --- | --- | --- | --- | --- | --- | --- | --- |
| AUC | 0.895 | 0.867 | 0.841 | 0.908 | 0.872 | 0.883 | 0.788 |
| Accuracy | 0.833 | 0.803 | 0.768 | 0.839 | 0.829 | 0.788 | 0.741 |
| Sensitivity | 0.811 | 0.829 | 0.769 | 0.813 | 0.947 | 0.800 | 0.900 |
| Specificity | 0.862 | 0.774 | 0.767 | 0.867 | 0.688 | 0.769 | 0.647 |
| Precision | 0.882 | 0.806 | 0.741 | 0.867 | 0.783 | 0.842 | 0.600 |
| F1 | 0.845 | 0.817 | 0.755 | 0.839 | 0.857 | 0.821 | 0.720 |
| **Avgerage** | 0.855 | 0.816 | 0.774 | 0.856 | 0.829 | 0.817 | 0.733 |

**Supplemental Table 7.** The baseline characteristics of the National University of Malaysia (UKM) Medical Centre PA cohort

| Predictors | All  N=27 | *WT- KCNJ5*  N= 17 | *KCNJ*5 mutation  N= 10 |
| --- | --- | --- | --- |
| Baseline Characteristics | | | |
| Gender (Male, n) | 16 (59.0%) | 11 (64.7%) | 5 (50%) |
| Age (year) | 47.63 (13.3) | 51.65 (12.6) | 40.8 (12.2) |
| BMI (kg/m^2^) | 26.56 (4.8) | 27.44 (4.7) | 25.07 (4.7) |
| SBP (mmHg) | 164.37 (24.4) | 158.94 (18.9) | 173.60 (30.0) |
| DBP (mmHg) | 94.00 (16.7) | 91.12 (14.2) | 98.90 (20.0) |
| Heart rate (beat per minute) | 82.11 (12.1) | 79.35 (13.3) | 86.85 (9.6) |
| Comorbidities | | | |
| Hypertension duration (year) | 5.84 (5.2) | 7.56 (5.5) | 2.77 (2.9) |
| Hypokalemia (n) | 11 (40.7%) | 8 (47.1%) | 3 (30.0%) |
| Laboratory Data | | | |
| Plasma Renin Activity (PRA) (ng/ml/hr) | 0.84 (0.65) | 0.81 (0.62) | 0.90 (0.77) |
| Serum Aldosterone (ng/dL) | 67.96 (39.6) | 63.89 (37.3) | 0.97 (0.50) |
| Serum Creatinine (mg/dL) | 0.96 (0.37) | 1.01 (0.51) | 77.11 (18.4) |
| Serum Na^+^ (mg/dL) | 140.37 (2.9) | 140.35 (2.8) | 140.40 (2.9) |
| Serum K^+^ (mmol/L) | 2.61 (0.59) | 2.82 (0.57) | 2.25 (0.46) |
| Serum Cortisol 8am (μg/dL) | 13.12 (4.88) | 12.59 (5.61) | 13.97 (3.42) |

**Summary of Malaysian Primary Aldosteronism (PA) data**

We enrolled 27 primary aldosteronism (PA) patients with (n = 10) or without (n = 17) the *KCNJ5* mutation. The diagnosis of PA was defined as having a positive confirmatory test such as Saline Suppression Test (SST) or Fludrocortisone Suppression Test (FST). Patients with informed consent were recruited from UKM Medical Centre from the year 2015 to 2020. The medical records of patients who had undergone adrenalectomy were taken from the Computed Tomography (CT) scan report and histopathology report. Fourteen features were collected including demographics, blood test results and clinical comorbidities. gDNA was extracted from the FFPE of APA samples using the ReliaPrep FFPE gDNA Miniprep System (Promega, USA) per the manufacturer’s instruction. The DNA sequence that encoded the selectivity filter region in *KCNJ5* was amplified using the AmpliTaq GoldTM Fast PCR Master Mix (ThermoFisher Scientific, USA) according to the manufacturer’s instructions using the following primers: *KCNJ5* Forward primer, GGA TTC CTT GTG TTG AAA ACC and *KCNJ5* Reverse primer, TCT TGG GCT GGC TGA TCT T. Polymerase chain reaction (PCR) products were then Sanger Sequenced commercially (Apical Scientific Sdn Bhd, Malaysia)

**Supplemental Table 8.** Ablation experiment for LightGBM hyperparameter setting.

| Tree setting (learning rate = 0.05, epoch = 100) | | | | | |
| --- | --- | --- | --- | --- | --- |
|  | **2** | **3** | **4** | **5** | **10** |
| AUC | 0.885 | 0.897 | **0.905** | 0.898 | 0.900 |
| Accuracy | 0.818 | 0.864 | **0.864** | 0.833 | 0.818 |
| Sensitivity | 0.857 | 0.886 | **0.886** | 0.857 | 0.829 |
| Specificity | 0.774 | 0.839 | **0.839** | 0.806 | 0.806 |
| Precision | 0.811 | 0.861 | **0.861** | 0.833 | 0.829 |
| F1 | 0.833 | 0.873 | **0.873** | 0.845 | 0.829 |
| Learning rate setting (tree = 4, epoch=100) | | | | | |
|  | 0.5 | 0.1 | **0.05** | 0.01 | 0.005 |
| AUC | 0.791 | 0.900 | **0.905** | 0.893 | 0.858 |
| Accuracy | 0.758 | 0.848 | **0.864** | 0.848 | 0.803 |
| Sensitivity | 0.800 | 0.829 | **0.886** | 0.914 | 0.857 |
| Specificity | 0.710 | 0.871 | **0.839** | 0.774 | 0.742 |
| Precision | 0.757 | 0.879 | **0.861** | 0.821 | 0.789 |
| F1 | 0.778 | 0.853 | **0.873** | 0.865 | 0.822 |
| Epoch setting (tree = 4, learning rate = 0.05) | | | | | |
|  | 10 | 50 | **100** | 150 | 200 |
| AUC | 0.891 | 0.896 | **0.905** | 0.904 | 0.905 |
| Accuracy | 0.833 | 0.848 | **0.864** | 0.879 | 0.848 |
| Sensitivity | 0.914 | 0.886 | **0.886** | 0.886 | 0.857 |
| Specificity | 0.742 | 0.806 | **0.839** | 0.871 | 0.839 |
| Precision | 0.800 | 0.838 | **0.861** | 0.886 | 0.857 |
| F1 | 0.853 | 0.861 | **0.873** | 0.886 | 0.857 |

References

1. Nayak S, Misra BB and Behera HS. Impact of data normalization on stock index forecasting. *International Journal of Computer Information Systems and Industrial Management Applications*. 2014;6:257-269.

2. Barceló JA. Chi‐square analysis. *The encyclopedia of archaeological sciences*. 2018:1-5.

3. Sterne JA, White IR, Carlin JB, Spratt M, Royston P, Kenward MG, Wood AM and Carpenter JR. Multiple imputation for missing data in epidemiological and clinical research: potential and pitfalls. *Bmj*. 2009;338.

4. Lin W-C and Tsai C-F. Missing value imputation: a review and analysis of the literature (2006–2017). *Artificial Intelligence Review*. 2020;53:1487-1509.

5. Nguyen CD, Carlin JB and Lee KJ. Model checking in multiple imputation: an overview and case study. *Emerging themes in epidemiology*. 2017;14:1-12.

6. Liu Z. A method of SVM with normalization in intrusion detection. *Procedia Environmental Sciences*. 2011;11:256-262.

7. Munkhdalai L, Munkhdalai T, Park KH, Lee HG, Li M and Ryu KH. Mixture of activation functions with extended min-max normalization for forex market prediction. *IEEE Access*. 2019;7:183680-183691.

8. Rodríguez P, Bautista MA, Gonzalez J and Escalera S. Beyond one-hot encoding: Lower dimensional target embedding. *Image and Vision Computing*. 2018;75:21-31.

9. Menard S. *Applied logistic regression analysis*: Sage; 2002.

10. Brefeld U and Scheffer T. AUC maximizing support vector learning. *Proceedings of the ICML 2005 workshop on ROC Analysis in Machine Learning*. 2005.

11. Chicco D and Jurman G. The advantages of the Matthews correlation coefficient (MCC) over F1 score and accuracy in binary classification evaluation. *BMC genomics*. 2020;21:1-13.

12. Briggs AH, Wonderling DE and Mooney CZ. Pulling cost‐effectiveness analysis up by its bootstraps: a non‐parametric approach to confidence interval estimation. *Health economics*. 1997;6:327-340.

13. Hooker S, Erhan D, Kindermans P-J and Kim B. A benchmark for interpretability methods in deep neural networks. *arXiv preprint arXiv:180610758*. 2018.

14. Rossi GP, Bernini G, Caliumi C, Desideri G, Fabris B, Ferri C, Ganzaroli C, Giacchetti G, Letizia C, Maccario M, Mallamaci F, Mannelli M, Mattarello MJ, Moretti A, Palumbo G, Parenti G, Porteri E, Semplicini A, Rizzoni D, Rossi E, Boscaro M, Pessina AC, Mantero F and Investigators PS. A prospective study of the prevalence of primary aldosteronism in 1,125 hypertensive patients. *J Am Coll Cardiol*. 2006;48:2293-300.

15. Abdelhamid S, Blomer R, Hommel G, Haack D, Lewicka S, Fiegel P and Krumme B. Urinary tetrahydroaldosterone as a screening method for primary aldosteronism: a comparative study. *Am J Hypertens*. 2003;16:522-30.

16. Lenzini L, Rossitto G, Maiolino G, Letizia C, Funder JW and Rossi GP. A meta-analysis of somatic KCNJ5 K+ channel mutations in 1636 patients with an aldosterone-producing adenoma. *The Journal of Clinical Endocrinology & Metabolism*. 2015;100:E1089-E1095.

17. Wong T-T and Yeh P-Y. Reliable accuracy estimates from k-fold cross validation. *IEEE Transactions on Knowledge and Data Engineering*. 2019;32:1586-1594.

18. Jung Y. Multiple predicting K-fold cross-validation for model selection. *Journal of Nonparametric Statistics*. 2018;30:197-215.
